# Factors affecting knowledge of autism spectrum disorder among pediatric residents in eastern China: a cross-sectional study

**DOI:** 10.1186/s12909-022-03770-4

**Published:** 2022-10-03

**Authors:** Chao Song, Lingling Wu, Yunxia Hong, Xiaoyang Chen, Zhiwei Zhu

**Affiliations:** 1 Department of Developmental and behavioral Pediatrics, the Children’s Hospital, Zhejiang University School of Medicine, National Clinical Research Centre for Child Health, Hangzhou, China; 2 Education Office, the Children’s Hospital, Zhejiang University School of Medicine, National Clinical Research Centre for Child Health, Hangzhou, China

**Keywords:** Autism spectrum disorder, Knowledge, Standardized training, Pediatric resident, China

## Abstract

**Background:**

There is a global increase in the prevalence of autism spectrum disorder (ASD). Early identification of ASD in children and intervention are key aspects in the management of ASD. However, early identification is partly dependent on knowledge on ASD among pediatricians. This study analyzed the extent of ASD knowledge and its underlying factors among pediatric residents in eastern China, to provide a reference for medical education reforms.

**Methods:**

The study employed the Knowledge about Childhood Autism among Health Workers questionnaire. A total of 138 pediatric residents participated in the survey. Descriptive statistics were used to describe demographic characteristics and the four domains of the questionnaire. Univariate analysis was employed to assess impacts of the demographic characteristics on the questionnaire scores. On the other hand, multivariate regression analysis was used to analyze the correlation between the participants’ demographic characteristics and the questionnaire scores.

**Results:**

The average ASD cognitive score of 138 respondents was 13.38 ± 4.48. The ASD cognitive scores in female pediatric residents were higher compared to that in males (*p* < 0.05). Residents who had obtained professional doctor qualification certificate were more than those without professional doctor qualification certificate (*p* < 0.05). The ASD knowledge in the group which did not have rotation in both departments was lower than in the group which had rotation in both departments (*p* < 0.05) as well as the group that had rotation in developmental and behavioral pediatrics department only (*p* < 0.05). Our multivariate linear regression model demonstrated significant statistical differences (*p* < 0.05), and showed that gender and systematic exposure to ASD knowledge had significant effects on cognitive scores (*p* < 0.05).

**Conclusion:**

Most participants had relatively low levels of awareness and knowledge about ASD, especially on ASD comorbidities and age of onset. Women, systematic learning of ASD knowledge in medical school, successful passing of the physician examination, and rotation in the developmental and behavioral pediatrics (DBP) department significantly influence the levels of ASD awareness and knowledge. It is, therefore, important to strengthen ASD education in medical students at the university level and make rotation in the DBP department a requisite for pediatric trainees.

## Introduction

Autism spectrum disorder (ASD) is a neurodevelopmental disorder characterized by social communication and social interaction impairments, as well as restricted and repetitive patterns of behavior, interests, or activities [1]. In recent years, the prevalence of autism has been on the rise worldwide. A 2020 US Centers for Disease Control and Prevention report showed that a prevalence of autism among 8-year-old children was 1.68% [2]. Surveillance data released in 2021 showed that the prevalence of ASD was 2.27%, demonstrating that 1 in 44 children are diagnosed with ASD (based on 11 surveillance sites in the United States, 8-year-old children) [3]. Latest survey results from 8 regions in China shows that the prevalence of autism in children aged 6–12 is 0.7% [4]. Thus, early interventions for children with ASD is quite important [5, 6]. Indeed, previous studies have demonstrated the effectiveness of early intensive behavioral interventions in the treatment of young children with ASD [7–9], which is associated with brain plasticity [10]. Since early intervention is the purposeof early identification of ASD, knowledge on ASD among pediatricians who are the first-contact medical professional for almost all children (including children with ASD) in early life play an important role in proper management of children with ASD [11, 12].

Although current mainstream guidelines indicate that ASD can be diagnosed as early as 18 months of age [13], children with ASD may show certain clinical symptoms as early as 13–14 months after birth, while some children can have symptoms as early as 8 months old [14]. Globally, the average age at diagnosis of ASD is 60.48 months, and the age at diagnosis of ASD in children under 10 years is 43.18 months [15]. Between 2004 and 2014, the age of diagnosis in children with ASD in the UK was 55 months, which has not decreased over the past decade [16]. In 2017, two surveys in the United States demonstrated that most children with ASD were diagnosed after 3 years old, and 1/3 to 1/2 were diagnosed after 6 years old [17]. Besides, delays in ASD diagnosis are more common in less developed countries [18, 19].

Previous studies have shown that the more the ASD knowledge in pediatricians and general practitioners, the more there are suspected ASD case-referrals to specialists [20]. Early identification largely depends on the ASD knowledge in clinicians and parents [21]. In China, the standardized training system for residents has been established and well implemented, which has improved both knowledge and skills [22, 23]. All medical clinicians, including pediatricians, should receive standardized training for residents lasting 3 years after graduating from medical school [24]. Residency training is a key phase in the development of a physician [25]. In these three years, residents will study in different departments in turn, and master the diagnosis and treatment norms of common diseases in corresponding specialties. After the standardized training, the pediatric residents will rarely have opportunities to systematically learn the diagnosis and treatment of neurodevelopmental disorders such as ASD, if no relevant knowledge has been acquired during this period.

Turkish scholars had used self-made questionnaires to investigate 45 pediatricians and 56 pediatric residents about ASD knowledge, beliefs and experiences. The results indicated a lack of awareness and knowledge of ASD among pediatric residents [26]. Due to the small sample size of this study and the differences in standardized training systems between China and Turkey, the above conclusion may not be applicable to Chinese pediatric residents. In addition, few studies have focused on the knowledge of ASD among pediatric residents. Therefore, evaluation of the current status and influencing factors of ASD cognition among Chinese pediatric residents would help in understanding and strengthening weaknesses in autism knowledge, and promote early identification and intervention in children with ASD, through medical education reforms in China.

## Methods

### Investigation site and participants

The study was conducted in January 2022, at the children’s hospital, Zhejiang University school of medicine, Zhejiang Province, Eastern China. The hospital is a national-level standardized training center for pediatric residents. A total of 138 pediatric residents participated in the survey. This study was approved by the Ethics Committee of the children’s hospital, Zhejiang University school of medicine (No. 2022-IRB-171).

### Questionnaire tool

The questionnaire consisted of two parts; the socio-demographic information and Knowledge about Childhood Autism among Health Workers (KCAHW) [27]. The sociodemographic questionnaire was used to collect information such as gender, age, hometown, annual income, education level, grade or learning experience in relevant departments. The KCAHW consisted of a total of 19 items which were divided into 4 knowledge domains: Domain 1 contained 8 items on social interaction; domain 2 contained 1 question addressing impairment in communication-language; domain 3 had 4 items which mainly included restricted, repetitive patterns of behavior, interests or activities; while domain 4 encompassed 6 items on co-morbidities and onset of childhood autism.

### Statistical analysis

The generated data were analyzed using Statistical Package for Social Sciences (SPSS) version 26. Descriptive statistics were used to define the demographic features. Each item had 3 options (yes, no and do not know), with a correct answer scored as one while an incorrect answer scored as zero. Scores per domain and total scores were calculated for resident physicians. Whether the differences between different groups are statistically significant were compared based on gender, age, education level, grade, qualification, branch of training, rotation experience or university major. The measurement data was expressed as a mean ± standard deviation, while the chi-square test was used to determine the equilibrium of the groupings for the basic situation of the data. If the normal distribution of the data in two groups was satisfactory, the t-test was used for inter-group comparison; otherwise, the MANN-Whitney test was employed. For more than two groups, if the data met the normal distribution and the variance was homogeneous, one-way analysis of variance (ANOVA) was used for inter-group comparison, while the LSD method was used for after-the-fact comparison; otherwise, the Kruskal-Wallis test was used. Except for the test level of the variance homogeneity test of 0.10, the rest of the test set the test level to 0.05, that is, a *p* < 0.05 indicated statistical significance.

## Results

### Socio-demographic characteristics and professional information of the respondents

A total of 138 resident physicians gave informed consent and participated in this survey, and all the questionnaires used were valid. The oldest respondent was 36 years old while the youngest one was 22 years old, with an average age of (26.25 ± 2.61) years old. 137 (99.3%) participants had a bachelor’s degree or above, while 108 (78.3%) had an annual income of less than 100,000 yuan. During medical school education, only 27 (17.4%) had at least one month of internship experience at the department of child health care or department of developmental and behavioral pediatrics (DBP). The basic socio-demographic characteristics and professional information of the respondents are shown in Table 1.

**Table 1:** Socio-demographic characteristics and professional information of the respondents

	Frequency	Percentage
**Gender**		
male	39	28.3%
female	99	71.7%
**Age**		
≤ 25 years old	61	44.2%
>25 years old	77	55.8%
**Hometown**		
Urban	52	37.7%
Rural	86	62.3%
**Education level**		
PhD or MD	12	8.7%
Master	38	27.5%
Bachelor and others	88	63.8%
**Grade**		
Grade 1	52	37.7%
Grade 2	35	37.0%
Grade 3	51	25.3%
**Annual income**		
<¥50,000	53	38.4%
¥50,000 ~ ¥100,000	55	39.9%
>¥100,000	30	21.7%
**Qualification**		
No physician qualification certificate	57	41.3%
Have physician qualification certificate	81	58.7%
**Branch of training**		
Pediatric Internal Medicine	118	85.5%
Pediatric Surgery	13	9.4%
others	7	5.1%
**Type of trainee**		
Formal staff	22	15.9%
Informal staff	74	53.6%
Professional master	42	30.4%
**Rotation experience**		
Department of Child Health Care	26	18.8%
Department of DBP	16	11.6%
Neither	82	59.4%
Both	14	10.2%
**University major**		
Pediatrics	59	42.8%
Others	79	57.2%
**Systematically study Child Health or DBP**		
Yes	16	11.6%
No	122	88.4%
**College internship experience**		
Internship experience in department of Child Health Care or department of DBP	24	17.4%
Neither	114	82.6%

### Pediatric residents’ knowledge of ASD and its influencing factors

Our analysis showed that the total KCAHW mean score was 13.38 ± 4.48, and the median score was 15. Compared with the other domains, the fourth domain which was related to comorbidity had relatively low score ( 3.62 ± 1.41) while the first domain had relatively high score (6.12 ± 2.26) (see Table 2). 71.7% of the respondents believed that children with ASD would not have a phenomenon of *staring into open space and not focusing on anything specific* (see Table 3). Nearly half of the respondents (45.7%) thought that children with ASD would not be associated with abnormal eating habits (see also Table 3).

**Table 2:** Pediatric residents’ score of KCAHW questionnaire (mean ± SD)

Domain	Min to max	Score	Median
Domain 1: Social interaction	0–8	6.12 ± 2.26	7.00
Domain 2: Communication and language	0–1	0.83 ± 0.38	1.00
Domain 3: Pattern of behavior	0–4	2.81 ± 1.29	3.00
Domain 4: Comorbidity and age of onset	0–6	3.62 ± 1.41	4.00
Total	0–19	13.38 ± 4.48	15.00

**Table 3:** Accuracy rate of the KCAHW questionnaire

Domain	Stem	Correct response	Incorrect responses
**Domain 1**			
	1–1 Marked impairment in use of multiple non-verbal behaviors such as eye to eye contact, facial expression, body postures and gestures during social interaction?	121(87.7%)	17(12.3%)
	1–2 Failure to develop peer relationship appropriate for developmental age?	121(87.7%)	17(12.3%)
	1–3 Lack of spontaneous will to share enjoyment, interest or activities with other people?	122(88.4%)	17(11.6%)
	1–4 Lack of social or emotional reciprocity?	124(89.9%)	14(10.1%)
	1–5 Staring into open space and not focusing on anything specific?	39(28.3%)	99(71.7%)
	1–6 The child can appear as if deaf or dumb?	99(71.7%)	39(28.3%)
	1–7 Loss of interest in the environment and surroundings?	106(76.8%)	32(23.2%)
	1–8 Social smile is usually absent in a child with Autism?	112(81.2%)	26(18.8%)
**Domain 2**			
	2 − 1 Delay or total lack of development of spoken language?	114(82.6%)	24(17.4%)
**Domain 3**			
	3 − 1 Stereotyped and repetitive movement (e.g. Hand or finger flapping or twisting)?	119(86.2%)	19(13.8%)
	3 − 2 May be associated with abnormal eating habit?	63(45.7%)	75(54.3%)
	3–3 Persistent preoccupation with parts of objects?	111(80.4%)	27(19.6%)
	3–4 Love for regimented routine activities?	95(68.8%)	43(31.2%)
**Domain 4**			
	4 − 1 Autism is Childhood Schizophrenia?	96(69.6%)	42(30.4%)
	4 − 2 Autism is an autoimmune condition?	98(71.0%)	40(29.0%)
	4 − 3 Autism is a neurodevelopmental disorder?	77(55.8%)	61(44.2%)
	4–4 Autism could be associated with Mental Retardation?	68(49.3%)	70(50.7%)
	4–5 Autism could be associated with Epilepsy?	38(27.5%)	100(72.5%)
	4–6 Onset of Autism is usually in( Neonatal age, infancy or childhood)?	123(89.1%)	15(10.9%)

The univariate analysis results ( see Table 4) were grouped according to whether the participants had rotation experience in the department of child health care or department of DBP, and the difference between at least two groups was statistically significant (F = 20.469, *p* < 0.001). The knowledge of ASD was lower in the group without rotation in both departments compared to the group with rotation in both departments (t=-42.211, *p* = 0.001) and the group with rotation in the DBP department only (t=-32.385, *p* = 0.001). Although the ASD knowledge in the group with no rotation was lower than in that with rotation in department of child health care only, the difference was not statistically significant.

**Table 4:** Univariate analysis of the ASD knowledge

	Scores	t	*p*
**Gender**		-3.54	0.001^*^
male(n = 39)	10.77 ± 6.10		
female(n = 99)	14.40 ± 3.14		
**Age**		-1.658	0.100
≤ 25 years old(n = 61)	12.64 ± 5.35		
>25 years old(n = 77)	13.96 ± 3.58		
**Hometown**		0.250	0.803
Urban(n = 52)	13.50 ± 4.51		
Rural(n = 86)	13.30 ± 4.49		
**Education level**		0.078	0.925
PhD or MD(n = 12)	13.83 ± 3.54		
Master(n = 38)	13.42 ± 4.53		
Bachelor and others(n = 88)	13.30 ± 4.48		
**Grade** ^**†**^		5.873	0.053
Grade 1(n = 52)	11.98 ± 5.43		
Grade 2(n = 35)	13.83 ± 4.49		
Grade 3(n = 51)	14.49 ± 2.79		
**Annual income**		0.653	0.722
<¥50,000(n = 53)	12.42 ± 5.68		
¥50,000 ~ ¥100,000(n = 55)	14.05 ± 3.15		
>¥100,000(n = 30)	13.83 ± 3.98		
**Qualification**		-2.427	0.017^*^
No physician qualification certificate(n = 57)	12.23 ± 5.25		
Have obtained physician qualification certificate(n = 81)	14.19 ± 3.67		
**Branch of training**		1.926	0.150
Pediatric Internal Medicine(n = 118)	13.64 ± 4.33		
Pediatric Surgery(n = 13)	12.62 ± 4.63		
others(n = 7)	10.43 ± 6.16		
**Type of trainee** ^**†**^		1.127	0.569
Formal staff(n = 22)	14.18 ± 3.45		
Informal staff(n = 74)	13.88 ± 3.61		
Professional master(n = 42)	12.07 ± 5.94		
**Rotation experience** ^**†**^		20.469	< 0.001^*^
Department of Child Health Care(n = 26)	14.50 ± 2.70		
Department of DBP(n = 16)	15.44 ± 2.16		
Neither(n = 82)	12.16 ± 5.13		
Both(n = 14)	16.07 ± 1.82		
**University major**		-0.594	0.554
Pediatrics(n = 59)	13.10 ± 5.27		
Others(n = 79)	13.58 ± 3.81		
**Systematically study Child Health or DBP**		-1.690	0.110
Yes(n = 16)	11.00 ± 6.19		
No(n = 122)	13.69 ± 4.14		
**College internship experience**		0.098	0.922
Internship experience in department of Child Health Care or department of DBP(n = 24)	13.46 ± 4.80		
Neither(n = 114)	13.36 ± 4.43		

† Kruskal-Wallis test, the rest underwent analysis of variance, * *p* < 0.05

In this study, gender, grade, age, hometown, education level, annual income, qualification, branch of training, type of trainee, rotation experience, university major, systematic study of Child Health or DBP or college internship experience were used as independent variables, while the cognitive score of ASD was taken as a dependent variable for multiple linear regression analysis ( see Table 5). The results showed that the regression model had significant statistical significance (F = 3.004, *p* = 0.001), and the independent variable could explain 16.0% of the changes in the cognitive scores. The significance test results in Table 6 showed that gender and systematic learning of ASD knowledge significantly influenced the changes of cognitive score (*p* < 0.05).

**Table 5:** Assignment of argument

Argument	
Gender	Male = 1, female = 2
Age	≤ 25 years old = 1, >25 years old = 2
Hometown	Urban = 1, Rural = 2
Education level	PhD or MD = 1, Master = 2, Bachelor and others = 3
Grade	Grade 1 = 1, Grade 2 = 2, Grade 3 = 3
Annual income	<¥50,000 = 1, ¥50,000 ~ ¥100,000 = 2, >¥100,000 = 3
Qualification	No physician qualification certificate = 1, Have obtained physician qualification certificate = 2
Branch of training	Pediatric Internal Medicine = 1, Pediatric Surgery = 2, Others = 3
Type of trainee	Formal staff = 1, Informal staff = 2, Professional master = 3
Rotation experience	Department of Child Health Care = 1, Department of DBP = 2, Neither = 3, Both = 4
University major	Pediatrics = 1, Others = 2
Systematically study Child Health or DBP	Yes = 1, No = 2
College internship experience	Internship experience in the department of Child Health Care or department of DBP = 1, Neither = 2

**Table 6:** Multivariate regression analysis of the ASD knowledge

Argument	β	t	*p*	VIF
(constant)	8.295	1.845	0.067	
Gender	2.949	3.511	0.001^*^	1.170
Age	-0.020	-0.021	0.983	1.886
Hometown	-0.153	-0.196	0.845	1.166
Education level	-0.065	-0.079	0.937	2.357
Grade	0.600	1.020	0.310	2.114
Annual income	-0.086	-0.118	0.906	2.511
Qualification	0.191	0.184	0.855	2.146
Branch of training	-0.814	-1.029	0.305	1.324
Type of trainee	-1.531	-1.825	0.070	2.550
Rotation experience	-0.109	-0.267	0.790	1.119
University major	0.171	0.165	0.869	2.148
Systematic study of Child Health or DBP	2.889	2.289	0.024^*^	1.336
College internship experience	-1.123	-1.018	0.311	1.429

* *p* < 0.05

## Discussion

Using the KCAHW questionnaire, the mean score for the study population was 13.38 ± 4.48 points, which suggested that there is a need to improve the ASD knowledge in Chinese pediatric residents. Our results were in sync with a previous study which evaluated ASD awareness and knowledge in child health care workers in Southwest China [28]. Pediatric residents or pediatricians in different countries had different levels of ASD knowledge. However, it is difficult to evaluate which country’s pediatric residents or pediatricians have better ASD knowledge because of the differences in questionnaires and demographic characteristics such as age and gender [26, 29]. In addition, child health care workers [30, 31], general practitioners [32], pediatric nurses [33], psychiatric nurses [34], rehabilitation therapists [35] and other professionals [36], as well as the general public [37] which include parents [38] and teachers [39], lack knowledge and awareness of ASD, which presents a major obstacle to early identification of ASD.

Factors such as gender, obtaining the doctor qualification certificate, and rotation experience in the departments of child health care and DBP significantly affect the pediatric residents’ knowledge of ASD. Medical residency training is a key transformation phase from being a medical student to a practicing physician [40]. During the standardized training period, the residents need to take many examinations, such as the qualification examination for medical practitioners, the graduation examination organized by each rotating department, the annual examination organized by the training base, and the completion examination organized by the national unified organization. Only by passing all the above examinations can they finally complete the standardized training of residents and obtain the certificate of qualification. The qualification examination for medical practitioners is a form of industry access adopted by countries around the world, through which one can obtain practicing rights. The examination covers ASD knowledge, and the students who pass the exam have more ASD knowledge than those who fail. In China, passing the medical practitioner qualification certificate exam is directly linked to successful completion of standardized training. Residents who have not pass the medical practitioner qualification certificate exam will be deemed unqualified for standardized training at the same time. This policy has promoted popularization of ASD knowledge among residents. ASD is a neurodevelopmental disorder [1], which is common in the department of DBP. The students who had rotated in the department of DBP had significantly higher ASD cognitive levels than those who had not rotated. Unfortunately, In the latest training plan issued by the state, rotation in the department of DBP is not mandatory, and even many pediatric training bases have not set up the DBP department. Only a few pediatric trainees had the opportunity to rotate in DBP for 1–2 months. It is important therefore to modify the standardized training program to ensure each student have opportunities to study in the department of DBP during the standardized training, to improve the pediatric resident’s understanding of ASD.

In agreement with previous evidence among medical and allied-medical practitioners [29], our results showed that domain 4 which is related to comorbidity (e.g. epilepsy) was the poorly understood. Many people with autism were initially referred for signs of co-occurring conditions rather than for the autism signs. Thus, identifying and treating co-occurring conditions is an essential component of early interventions [41]. The survey demonstrated that about half of the respondents believed that ASD would not be associated with eating behavior. In fact, about 70% of children with ASD have feeding and/or eating behavior problems, of which 36% were serious problems [42]. Abnormal eating habits is a manifestation of narrow interest and repetitive stereotyped behavior in children with ASD. The above contents should be the basis of teaching in the standardized training protocols of pediatric residents.

Furthermore, by comparing our findings with existing studies, it was unfortunately found that no studies consistent with the participants and survey tools of this paper were found up to this point. Only the KCAHW survey was used for other respondents, such as pediatric nurses. Alternatively, a self-made questionnaire (with very different items from the KCAHW questionnaire) was used to survey pediatric residents. The studies that have been conducted and published using the KCAHW questionnaire have not analyzed the correct or incorrect rates of each item in detail. Therefore, it is difficult to compare the results of the above studies with the results of our study.

To control for possible confounding, findings from our multivariate regression analysis model which could reduce the effect of confounding factors showed that in addition to gender, systematic study of child health care or developmental behavioral pediatrics in the medical school period can also affect the cognition of ASD by pediatric residents. Most pediatricians in China did not graduate from pediatrics medicine, but from clinical medicine, whose courses hardly include ASD. Other studies have demonstrated similar results, indicating that medical school education on ASD is not sufficient [43–45]. Pediatrics is a compulsory course for clinical medicine majors. Thus, incorporating ASD content into the pediatrics teaching of clinical medicine students may help deepen the understanding of ASD among doctors. Interestingly, in both univariate and multivariate regression analysis, gender was shown to significantly affect the perception of ASD among pediatric residents. Whether this outcome was related to the higher proportion of female pediatricians or caused by other reasons requires further evaluation.

Although our study yielded major findings, the sample size was small and was based on a single-center study. There is a need for a multi-center survey on the ASD knowledge in standardized training in national, provincial, and municipal training centers in different regions of China.

## Conclusion

Taken together, our study showed that most participants had relatively low levels of awareness and knowledge about ASD, especially on ASD comorbidities and age of onset. Women, systematic learning of ASD in medical school, passing the physician examination, and rotations in the department of DBP are significant contributors to higher levels of ASD awareness and knowledge. It is important to strengthen the ASD education in medical students at the university level, and incorporate the DBP department in standardized training rotations in pediatrics.

## Data Availability

The datasets used and/or analyzed during the current study are available from the corresponding author on reasonable request.
